# 

**Published:** 1912-10-15

**Authors:** 


### ANNOUNCEMENTS.

Union Meeting of the 7th and 8th District Dental Societies of New York State.

On November 14th, 15th and 16th, 1912, there will be held what is confidently expected will be the largest meeting of the dentists of eastern, central and western New York as well as a number from western Pennsylvania and adjacent points in Canada. As it is two years since the 7th and 8th District Dental Societies have held a Union Meeting, every member of these two societies and a great number from the 5th District Dental Society of the same state are expected to attend and help promote the interests of this meeting.

Prominent essayists and able clinicians have been secured to provide those who attend with plenty to think about on very interesting subjects and helpful assistance they can use in their everyday practice.

There will also be an extraordinarily large exhibit of the different manufacturers from all over the country, who will bring the latest scientific methods and appliances for demonstration by men who are thoroughly conversant with the needs and requirements of dentists, and every dentist within convenient distance should make it a point to be at this important meeting where there will be so much to learn and see which will be useful in helping him to become a better practitioner.

Business Committee:

E. G. Link, (*Chairman.*)

THE THOS. W. EVANS MUSEUM AND DENTAL INSTITUTE. By a formal agreement executed on June 15, 1912, by the Trustees of the University of Pennsylvania and The Thomas W. Evans Museum and Institute Society, a co-operative affiliation is now established between the respective corporations whereby the resources of both will be utilized in carrying out the intent and purposes expressed in the will of the late Dr. Thomas W. Evans in which he directed that the residue of his estate be applied to the creation of a Dental Educational Institution to be located at the northwest corner of Spruce and Fortieth Streets, in the City of Philadelphia, and to be carried on "As such institutions of learning are now conducted in Philadelphia and not inferior to any already established." Plans for the new Dental Institute which will house the School of Dentistry of the University of Pennsylvania have been completed, and as the work of construction will begin at once, it is expected that the new building will be ready for occupancy at the session beginning October 1, 1913.

The Evans Museum, which will occupy the east hall of the Spruce Street wing, will be as nearly fire and burglar proof as modern science can make it.

In the scheme of educational work of the Institute, provision has been made for the undergraduate training for the dental degree as required for legal qualification for the practice of dentistry; post-graduate instruction on the elective system, and opportunities and facilities for scientific research in dental subjects.

In consequence of the affiliated interests of the two institutions, the students of the Evans Institute will be accorded all of the advantages and university relationships now enjoyed by students of the Dental School of the University, including the resources of the University for furnishing instruction in the fundamental medical and dental departments, its social, educational and athletic advantages.

The students of the Dental School of the University will, in return for these concessions, enjoy the advantages of the new building which will be erected by the Evans Institute Society, and its material resources for the conduct, maintenance and improvement of the same.

It is the expectation that, when completed, it will constitute the largest, most complete and efficiently organized dental educational institution in existence in the several phases of undergraduate, post-graduate and dental research work.

---

CHICAGO DENTAL SOCIETY. The officers of the Chicago Dental Society are planning a large celebration for Friday and Saturday January 31st, and February 1st, 1913. The program includes two days of clinics by selected men from all parts of the country, one evening of papers by men of international reputation, concluding the two days meeting with a testimonial banquet to our esteemed confrere, Dr. Truman W. Brophy, of Chicago.

The dentists of Chicago will make every effort to see that the entire program will eclipse all former meetings. Any dentist who has a new or interesting clinic to give at this meeting is cordially invited to correspond with the Chairman of the Clinic Committee.

DR. FRED. W. GETHRO,
917 Marshall Field Bldg.,
Chicago, Ills.

---

INSTITUTE OF DENTAL PEDAGOGICS. The next annual meeting of the Institute of Dental Pedagogics will be held in Pittsburgh, Pa., January 28, 29, 30, 1913. An unusually interesting program has been arranged and no progressive dental teacher can afford to miss this meeting.

FRED. W. GETHRO, *Secretary*.

